# Diverse activation patterns during persistent atrial fibrillation by noncontact charge‐density mapping of human atrium

**DOI:** 10.1002/joa3.12361

**Published:** 2020-05-20

**Authors:** Rui Shi, Zhong Chen, Charlie Butcher, Junaid AB Zaman, Vennela Boyalla, Yi Kan Wang, Omar Riad, Anitha Sathishkumar, Mark Norman, Shouvik Haldar, David G Jones, Wajid Hussain, Vias Markides, Tom Wong

**Affiliations:** ^1^ Department of Cardiovascular Medicine The First Affliated Hospital of Xi'an Jiaotong University Xi'an China; ^2^ Heart Rhythm Centre The Royal Brompton and Harefield NHS Foundation Trust National Heart and Lung Institute Imperial College London London UK; ^3^ Auckland Bioengineering Institute University of Auckland Auckland New Zealand

**Keywords:** activation pattern, atrial fibrillation, localized irregular activation, localized rotational activation, noncontact charge‐density mapping

## Abstract

**Background:**

Global simultaneous recording of atrial activation during atrial fibrillation (AF) can elucidate underlying mechanisms contributing to AF maintenance. A better understanding of these mechanisms may allow for an individualized ablation strategy to treat persistent AF. The study aims to characterize left atrial endocardial activation patterns during AF using noncontact charge‐density mapping.

**Methods:**

Twenty‐five patients with persistent AF were studied. Activation patterns were characterized into three subtypes: (i) focal with centrifugal activation (FCA); (ii) localized rotational activation (LRA); and (iii) localized irregular activation (LIA). Continuous activation patterns were analyzed and distributed in 18 defined regions in the left atrium.

**Results:**

A total of 144 AF segments with 1068 activation patterns were analyzed. The most common pattern during AF was LIA (63%) which consists of four disparate features of activation: slow conduction (45%), pivoting (30%), collision (16%), and acceleration (7%). LRA was the second‐most common pattern (20%). FCA accounted for 17% of all activations, arising frequently from the pulmonary veins (PVs)/ostia. A majority of patients (24/25; 96%) showed continuous and highly dynamic patterns of activation comprising multiple combinations of FCA, LRA, and LIA, transitioning from one to the other without a discernible order. Preferential conduction areas were typically seen in the mid‐anterior (48%) and lower‐posterior (40%) walls.

**Conclusion:**

Atrial fibrillation is characterized by heterogeneous activation patterns identified in PV‐ostia and non‐PV regions throughout the LA at varying locations between individuals. Clinical implications of individualized ablation strategies guided by charge‐density mapping need to be determined.

## Graphical abstract

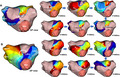

## 1 INTRODUCTION

The mechanisms of persistent atrial fibrillation (AF) are not well understood. Focal activation from the muscle sleeves within the pulmonary veins (PVs) is considered an important and common trigger of paroxysmal AF.^1^ However, the optimal ablation strategy to treat persistent AF remains unclear.^2^ While PV isolation alone appears equivalent to more extensive ablation strategies in recent randomized trials, there is a ceiling of efficacy which is lower than that for paroxysmal AF ablation.^3^, ^4^ Recent research efforts have been focused on the identification of patient‐specific electrical triggers/drivers of persistent AF outside the PVs to devise a more effective treatment strategy.

Narayan et al proposed that focal impulses and localized rotational activation (rotors) functioned as maintainers of AF.^5^ However, others have observed multiple wavefronts being the most common activation pattern seen during either noninvasive body surface mapping or contact epicardial mapping of AF, and therefore reasoned that rotors might not play such a critical role.^6^, ^7^, ^8^

A novel noncontact charge‐density mapping system (Acutus Medical, Carlsbad, CA) provides the opportunity to assess global endocardial atrial activation during AF with improved accuracy from ultrasound acquired anatomy/geometry and simultaneous assessment of cardiac activation. Charge density represents a continuous, tissue‐level distribution of the actual dipole sources of electric charge that exist at the cellular level and generate the cardiac potential field (distribution of voltage) throughout the torso volume.^9^ Theoretically, noncontact charge‐density mapping provides a more localized, higher‐resolution portrayal of complex and irregular activation patterns than voltage‐based mapping and is not affected by the same limitations as contact mapping.

This study aimed to use this novel charge‐density mapping technology to identify diverse activation patterns and their distributions in the left atrium (LA) during persistent AF.

## 2 METHODS

### 2.1 Patients

Twenty‐five consecutive patients (mean age: 65 ± 12 years; 18 males) with persistent AF undergoing de novo ablation therapy using charge‐density mapping were enrolled. Patients with prior cardiac surgical intervention, left atrial size > 50 mm, and any history of thromboembolic events were excluded. All patients gave written informed consent as part of their clinical procedure. The study was approved by the national ethics committee (NHS Health Research Authority).

### 2.2 Noncontact charge‐density mapping

Voltage and charge density are intrinsically linked because the magnitude of voltage at any point is a summation across all dipolar charge that is directly proportional to the strength of each charge and inversely proportional to the distance to each charge. The charge‐density mapping system provides static and dynamic three‐dimensional (3D) maps of electrical activation across an ultrasound‐acquired cardiac chamber surface.^9^, ^10^ Further details of charge‐density mapping are discussed in the supplementary methods. Our validation data showed the noncontact electrograms correlated well with contact electrograms in terms of morphology and timing difference when the centre of the noncontact catheter was ≤ 40 mm from the atrial endocardial wall.^11^

A 10F, 48‐pole noncontact mapping catheter (AcQMap catheter, Acutus Medical, Carlsbad, CA) was used to collect electroanatomical data. The mapping catheter when deployed is a 25‐mm‐diameter spheroid with six splines, including eight engineered biopotential electrodes and eight ultrasound transducers alternately placed on each spline. Ultrasound is used to reconstruct the 3D endocardial surface of the chamber. Global unipolar noncontact intracardiac potentials are sensed from the 48 biopotential electrodes of the catheter and are processed by an inverse solution to derive the dipolar charge sources at the endocardial surface (the cause of the potential field, measured as charge density). The corresponding waves of activation are displayed across the reconstructed 3D surface through time, as a propagation history map.

### 2.3 The electrophysiology procedure

A deflectable decapolar catheter was inserted into the coronary sinus and a quadripolar catheter was positioned in the inferior vena cava, with one electrode connected as a unipolar reference. The noncontact mapping catheter was introduced to the LA via a 12F deflectable sheath (AcQGuide sheath, Acutus Medical, Carlsbad, CA) following transseptal access. The LA geometry was ultrasonically constructed by rotating the mapping catheter after deployment in the centre of the atrial chamber. Technical details about clinical data acquisition have been described previously.^9^

### 2.4 AF mapping and data collection

In each patient, temporal segments of AF were selected for off‐line analysis from within at least five of the longest R‐R intervals identified in each 30‐second preablation recording. This avoided potential confounding distortion imposed by the QRS‐T subtraction algorithm upon reconstruction of low‐voltage atrial electrogram patterns. In each AF segment, charge‐density activation maps were generated and AF activation patterns were characterized.

**FIGURE 1:**
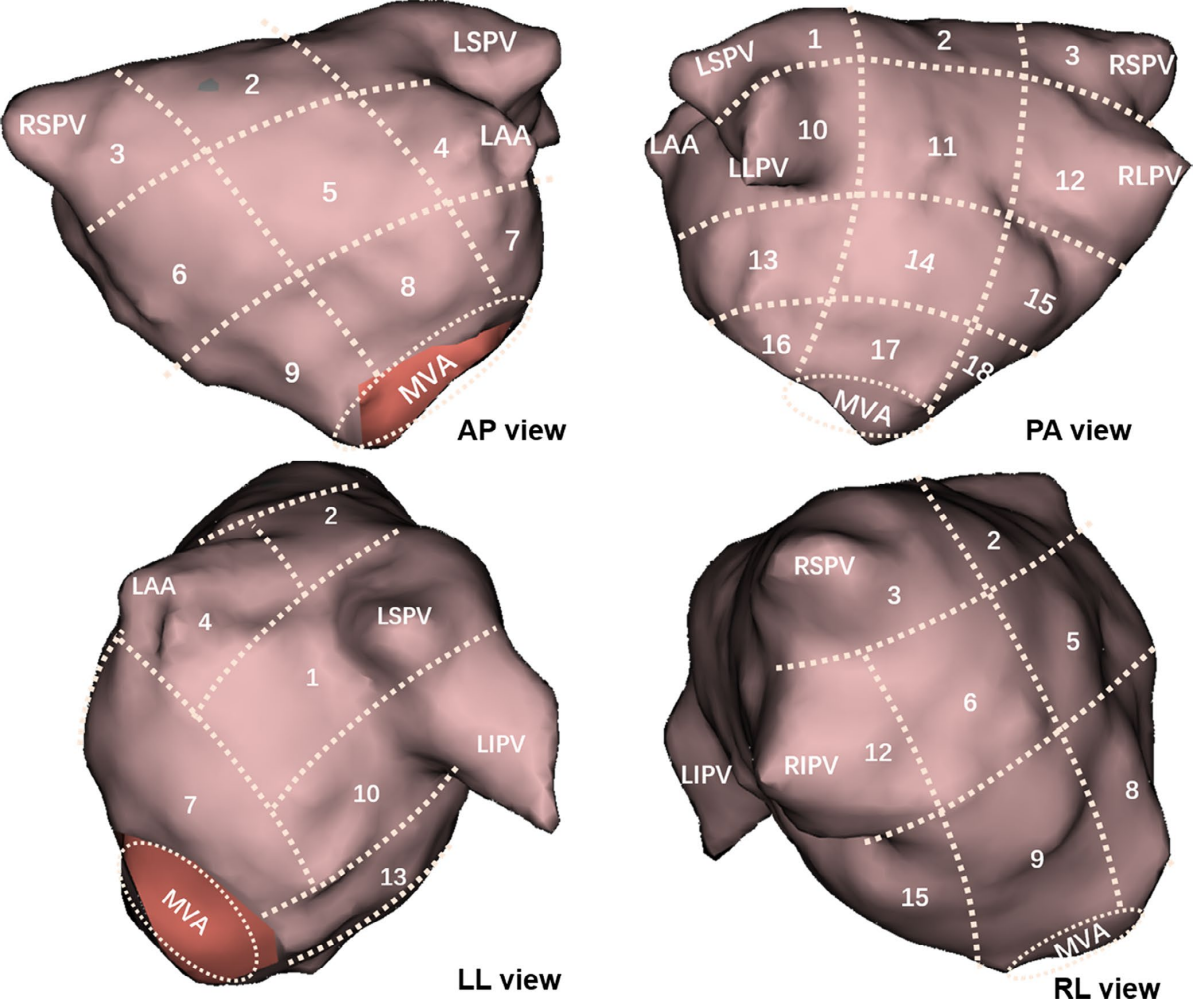
Anatomical regions of the left atrium. The 3D geometry of the LA is divided into 18 regions for demarcating the distribution of activation patterns and are displayed in the anteroposterior (AP) view, posteroanterior (PA) view, left lateral (LL) view, and right later (RL) view, respectively. Numbered regions 1, 3, 10, and 12 represent pulmonary vein/ostium; the number 4 represents the left atrial appendage (LAA); and the remaining numbered regions include the roof, floor, anterior wall, posterior wall, and lateral and septal wall of the LA, respectively

The LA was systematically divided into 18 regions, as illustrated in Figure 1, including the PV ostia, left atrial appendage, roof, floor, septum, lateral, and anterior and posterior walls. Any activation pattern appearing more than twice, consecutively, during each segment was counted as “repetitive”. A pattern which appeared equally on the border of two regions was attributed to both regions.

### 2.5 Activation pattern classification

Activation patterns were classified into three categories: focal centrifugal activation (FCA), localized rotational activation (LRA), and localized irregular activation (LIA).^9^, ^12^ The number, location, and direction of wavefront propagation were noted for each activation pattern, with directionality determined visually. The activation pattern within each numbered LA region can be dominated by either one or multiple wavefronts (such as LIA).

#### 2.5.1 Focal centrifugal activation

**FIGURE 2:**
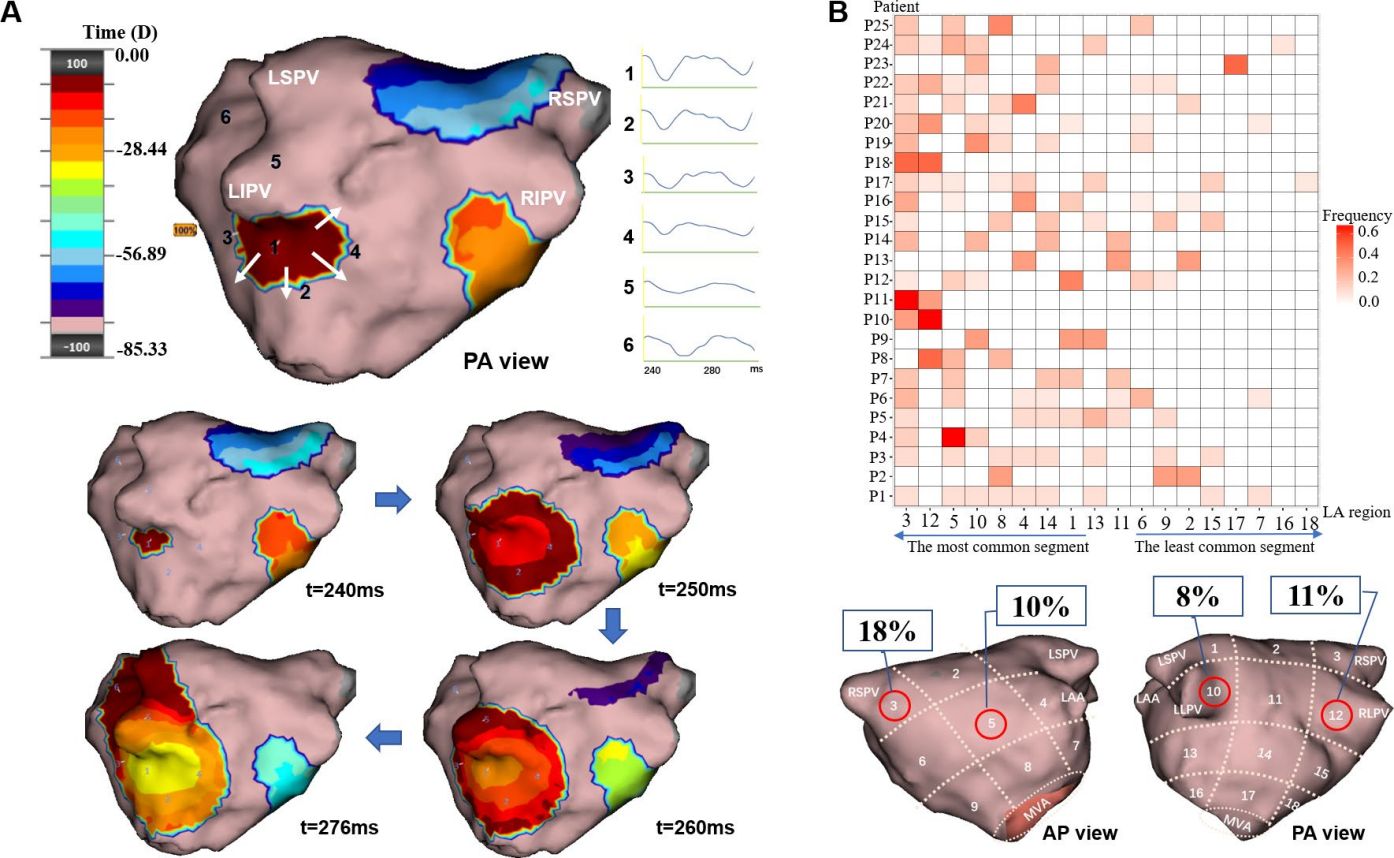
Focal centrifugal activation (FCA) identified by charge‐density mapping and the “Heat‐map” of FCA for each patient. Panel A shows an example of a FCA involving the left inferior pulmonary vein (LIPV) (Movie A in the online Data Supplement). White arrows show the centrifugal path of the propagation at the LIPV. The virtual electrograms taken at the site of focal source (1) and the following sequential activation sites (2‐6) are shown beside the upper activation map. The propagation map is color‐coded using the standard “thermal” scale to represent a “windowed‐history” of activation‐time across the endocardium with the leading edge of the wavefront shown in maroon. The time (milliseconds) at the bottom of each snapshot represents the moment when the snapshot is taken. Panel B shows the “Heat‐map” illustrating the frequency and distribution of the activation pattern in each patient. The *x*‐axis denotes the order from the most to the least common LA region where the activation pattern is observed. The intensity of the color bar reflects how frequent the activation pattern is observed, which is measured by the percentage of activation among the total patterns observed in individual patient. The most common regions on the anterior and posterior walls are labeled in the LA region map below

A discrete early activation within the mapping region with radial propagation to the periphery (Figure 2A).

#### 2.5.2 Localized rotational activation

**FIGURE 3:**
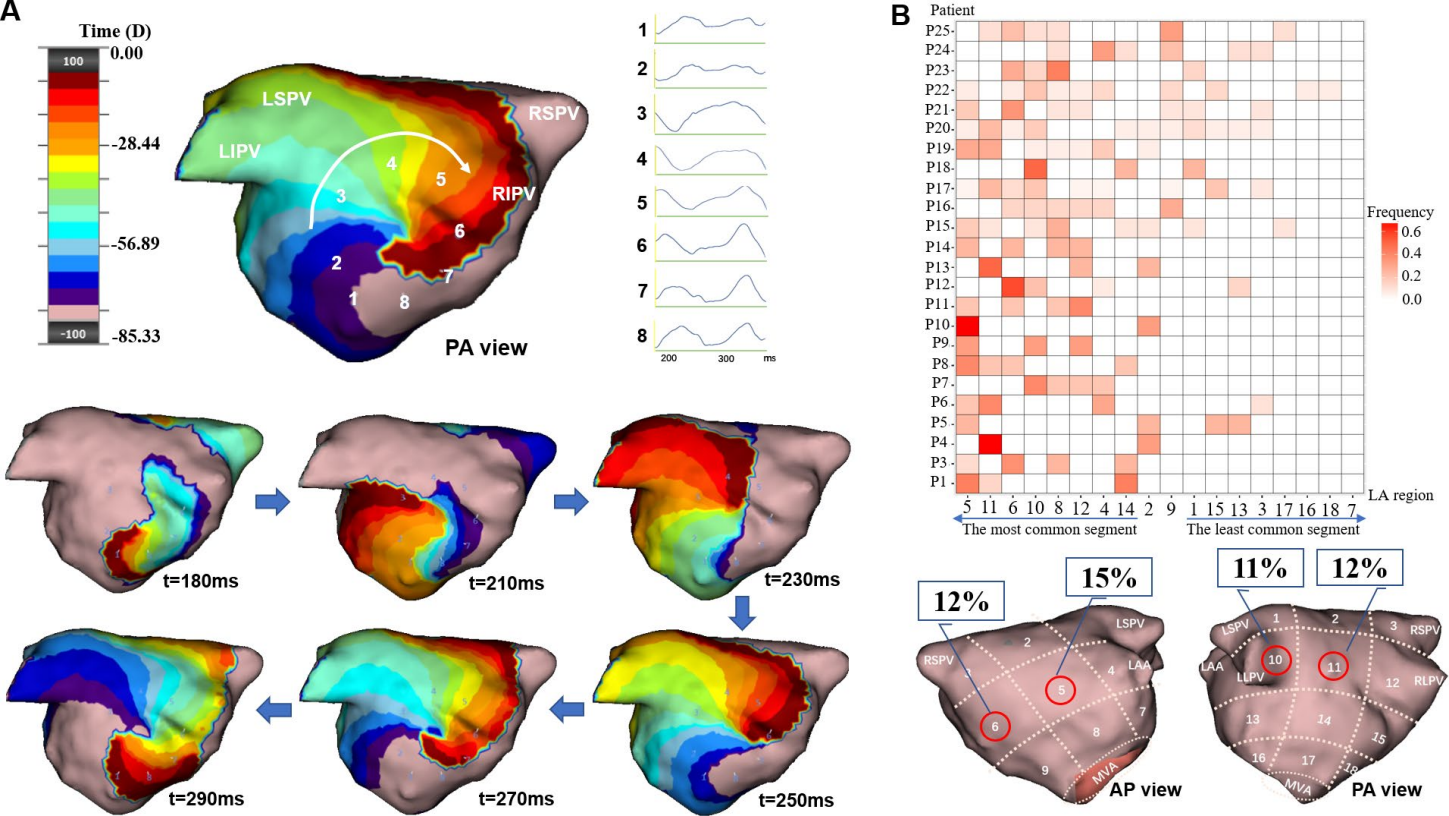
Localized rotational activation (LRA) identified by charge‐density mapping and the “Heat‐map” of LRA for each patient. Panel A shows an example of LRA on the lower‐posterior wall of the LA in a patient with persistent AF (Movie B in the online Data Supplement). The white arrow delineates the rotational direction of the LRA. The virtual electrograms taken along the path of LRA (1‐8) are shown, respectively. The time (milliseconds) at the bottom of each snapshot represents the moment when the snapshot is taken. Panel B shows the “Heat‐map” illustrating the frequency and distribution of LRA in each patient. See Figure 2 legends for details of the figure annotations

A spiraling wave of activation (rotation of ≥ 270°) centered on a confined zone located inside the mapping region (Figure 3A).

#### 2.5.3 Localized irregular activation

**FIGURE 4:**
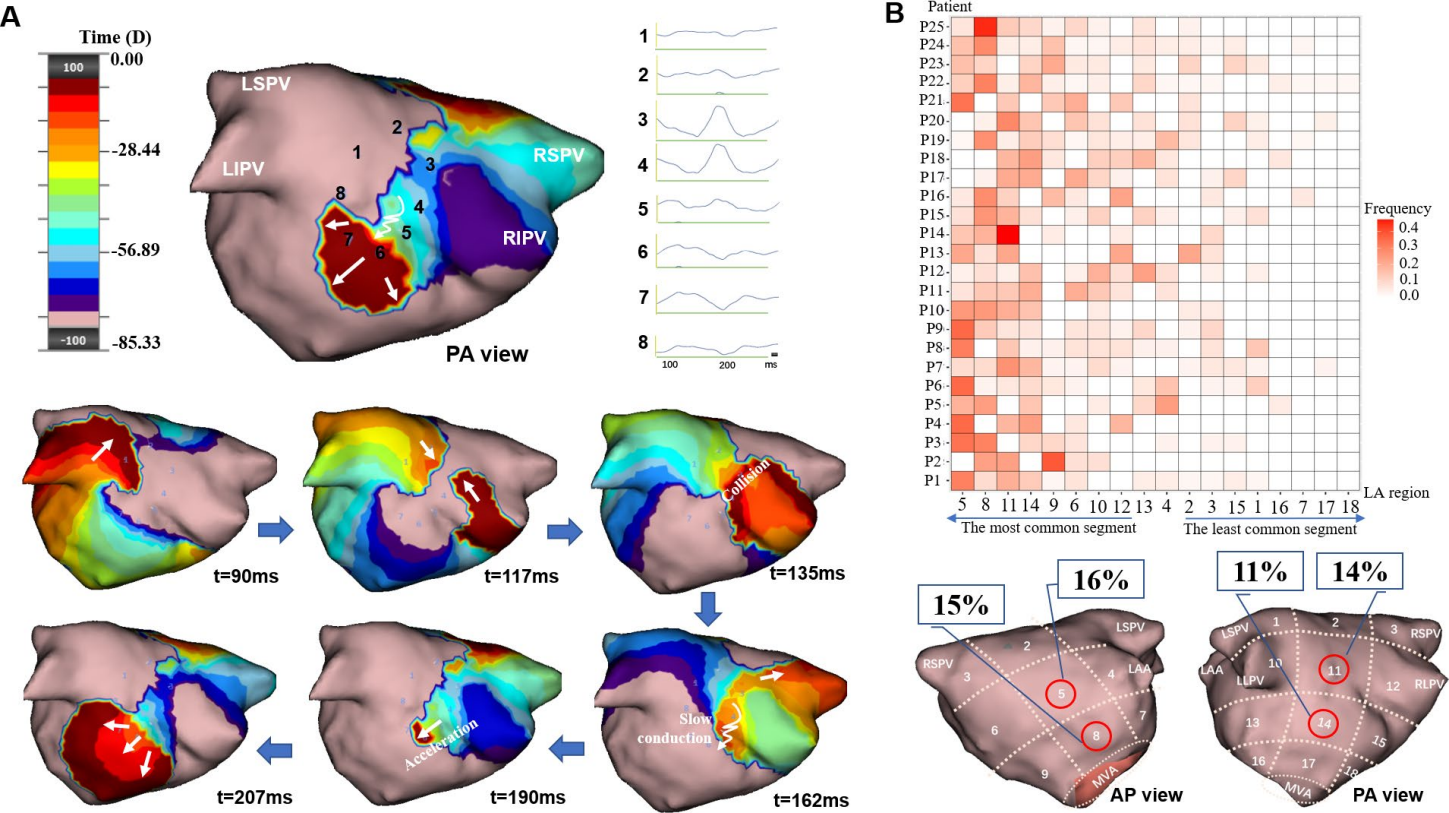
Localized irregular activation (LIA) identified by charge‐density mapping and the “Heat‐map” of LIA for each patient. Panel A shows an example of LIA on the posterior wall of LA in a patient with persistent AF (Movie C in the online Data Supplement). The white arrows show the activation direction of the LIA. A pivot can be seen first, then wavefront collision, followed by a slow conduction, and then acceleration through a gap at the posterior wall. The virtual electrograms taken along the path of LIA (1‐8) are shown, respectively. The time (milliseconds) at the bottom of each snapshot represents the moment when the snapshot is taken. Panel B shows the “Heat‐map” illustrating the frequency and distribution of LIA in each patient. See Figure 2 legends for details of the figure annotations

A localized activation with isthmus‐like entry and exit across a confined zone, and pivoting or unsynchronized propagation in at least two directions in the adjacent region surrounding the zone (Figure 4A).

**FIGURE 5:**
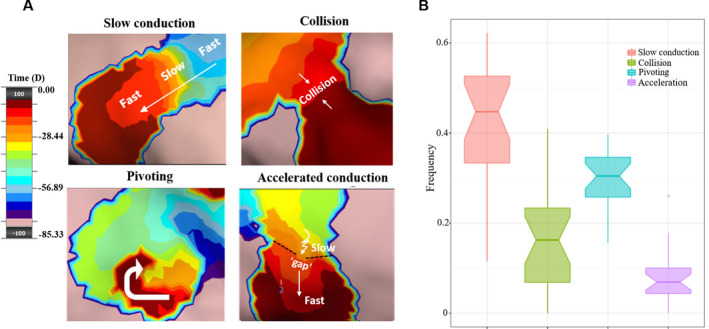
Proportion of four components of localized irregular activation. Panels A shows the four components of localized irregular activation (LIA). The propagation map is color coded using the standard “thermal” scale to represent a “windowed‐history” of activation time across the endocardium with the leading edge of the wavefront shown in maroon. The box plots in Panel B illustrate the proportion of each component of LIA observed. The *x*‐axis denotes four types of components; the y‐axis denotes the percentage of each component in all cases

Four distinct features of LIA (Figure 5A) include:

1. Slow conduction: deceleration of an activation wave within a confined zone.
2. Collision: fusion of two or more waves of activation within a confined zone.
3. Pivoting: partial rotation of an activation wave around a confined zone, with an angular sweep of less than 270°.
4. Accelerated conduction: acceleration of an activation wave after breaking out of a gap in a confined zone.

### 2.6 Preferential conduction area

Preferential conduction area was defined by the presence of two or more repeated or multiple patterns of conduction during the recording period. The spatial distribution of preferential conduction areas was assessed by visual analysis of the AF activation maps.

### 2.7 Statistical analysis

Distributions of all quantitative data were evaluated using the Kolmogorov‐Smirnov test. Continuous and normally distributed data were represented by mean ± standard deviation (SD). Unpaired *t* tests were used to compare two groups. Nonnormally distributed data were expressed as median (interquartile range IQR) and Mann‐Whitney U test was used to compare two groups. A *P* value <.05 was considered significant. All statistical analysis was performed using SPSS software version 22.0 (SPSS, Chicago, IL).

## 3 RESULTS

### 3.1 Patient demographics

The mean age of patients was 65 ± 12 years (72% male) with a median AF duration of 10 (IQR: 6‐12) months. The mean LA diameter was 44 ± 6 mm. Patient baseline demographics are shown in Table 1.

**Table 1:** Patient demographics

Total	N = 25
Age, years	65 ± 12
Male	18 (72%)
Median duration of AF, months	10 (IQR: 6‐12)
BMI, kg/m^2^	28 ± 4
Comorbidities	
Hypertension	10 (40%)
Diabetes mellitus	5 (20%)
Heart failure, NYHA ≥ 2	9 (36%)
Antiarrhythmic medications	
Beta‐blockers	23 (92%)
Flecainide	4 (16%)
Amiodarone	3 (12%)
Anticoagulation	
Warfarin	4 (16%)
Apixaban	10 (40%)
Dabigatran	5 (20%)
Rivaroxaban	6 (24%)
Previous failed DCCV	22 (88%)
LA diameter, mm	44 ± 6
LVEF, %	55 (IQR: 35‐60)
CHA2DS2‐VASc > 2	16 (64%)

Note

Values are mean ± SD, n (%), or median (interquartile range, IQR).

Abbreviations: AF, atrial fibrillation; BMI, body mass index; DCCV, direct current cardioversion; LA, left atrial; LVEF, left ventricular ejection fraction.

### 3.2 Activation patterns

A total of 144 AF segments were selected from R‐R intervals with a median duration of 1090 (IQR: 932‐1292) ms Charge‐density activation maps were generated from these segments and 1068 discrete instances of the three defined activation patterns were identified. The average distance from the centre of noncontact catheter to the LA anatomy was 29.7 ± 10.1 mm. On average, 43 ± 17 activation patterns were identified per patient, with 7 ± 1 activation patterns per map.

**FIGURE 6:**
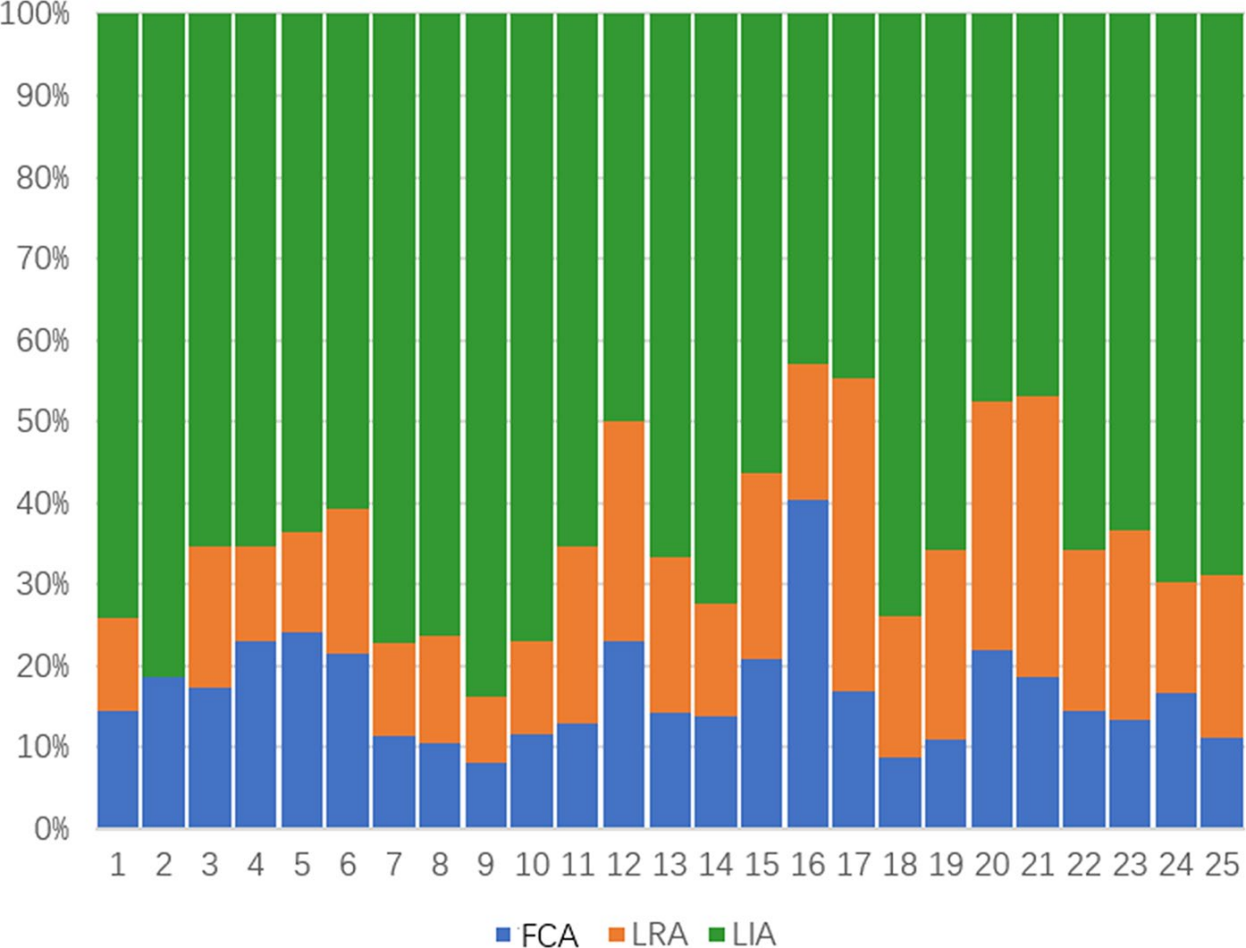
Percentage of atrial fibrillation activation patterns for each patient. The x‐axis denotes the patient number; the y‐axis denotes the frequency of each activation patterns. FCA = focal centrifugal activation; LIA = localized irregular activation; LRA = localized rotational activation

AF activation maps showed continuous and highly dynamic combinations of FCA, LRA, and LIA at discrete locations in all regions of the LA. These patterns temporally transitioned from one to the other without a discernible order, except for one patient (Patient 2), in whom LRA was not observed. The overall frequency distribution of activation patterns was as follows: (i) FCA = 17%; (ii) LRA = 20%; and (iii) LIA = 63%. The percentage of these AF patterns for each patient is displayed in Figure 6.

### 3.3 Focal centrifugal activation

Focal with centrifugal activation was identified in all patients and at discrete locations in all 18 LA regions. Focal activations were all short lived. The average number of FCA per R‐R interval was 2 ± 1. The mean number of focal sites per patient was 4 ± 2. Figure 2A shows the propagation of a FCA initiating near the left inferior PV. Figure 2B displays the frequency distribution of FCA in each patient. The most common regions of FCA were the right superior PV/ostium (18%) and right inferior PV/ostium (11%). Overall, the PV ostia had significantly more FCA than the non‐PV regions (10% vs 4%, *P* = .04).

### 3.4 Localized rotational activation, LRA

Localized rotational activation was seen in all patients except one (Patient 2). Both clockwise (51%) and counterclockwise (49%) rotation were observed in 17/18 LA regions. The average number of LRA within each AF segment was 2 ± 1. Continuous repetitive LRA (≥2 cycles) was observed in 8/25 patients with a median multirotational duration of 275 (IQR: 238‐380) ms. These LRAs periodically reformed at the same anatomic location in each patient. Figure 3A shows the propagation map of a LRA centered at the lower‐posterior wall. Figure 3B displays the frequency distributions of LRA in each patient. The most common regions of LRA were mid/septal‐anterior wall (15%; 12%) and mid‐posterior wall (12%).

### 3.5 Mode of LRA initiation

More than half of LRA (53%) appeared to initiate from a single broad wave tail that coalesced from multiple components of LIA, including slow conduction, pivoting, collision, and acceleration. Conversely, sustained LRAs (24%) broke and degenerated into LIA, while 42% of the LRAs were not associated with either FCA or LIA.

### 3.6 Localized irregular activation

Continuously varying LIA was observed in all patients, in all 18 LA regions with average of 5 ± 2 LIAs identified per AF segment. Figure 4A shows the propagation map of a LIA on the mid‐posterior wall. Figure 4B displays the frequency and distributions of LIA in each patient. The most common regions of LIA were mid‐ and lower‐anterior wall (16%; 15%) and mid‐ and lower‐posterior wall (14%; 11%).

Continuously varying propagation components of LIA were observed in all activation maps, with multiple combinations of the four components (Figure 5A). The most common component was slow conduction (45%); followed by pivoting (30%), collision (16%), and acceleration (7%) (Figure 5B). These four features, which are distinct patterns not present in LRA and FCA, were not always present in LIA simultaneously.

In a subgroup of 16 patients, 118 AF segments of RA activation were analyzed. Similar patterns of activations were observed. The most frequent pattern was LIA (75.6%), followed by FCA (21.1%) and LRA (3.1%) at posterior wall & septal junction.

### 3.7 Preferential conduction area

**FIGURE 7:**
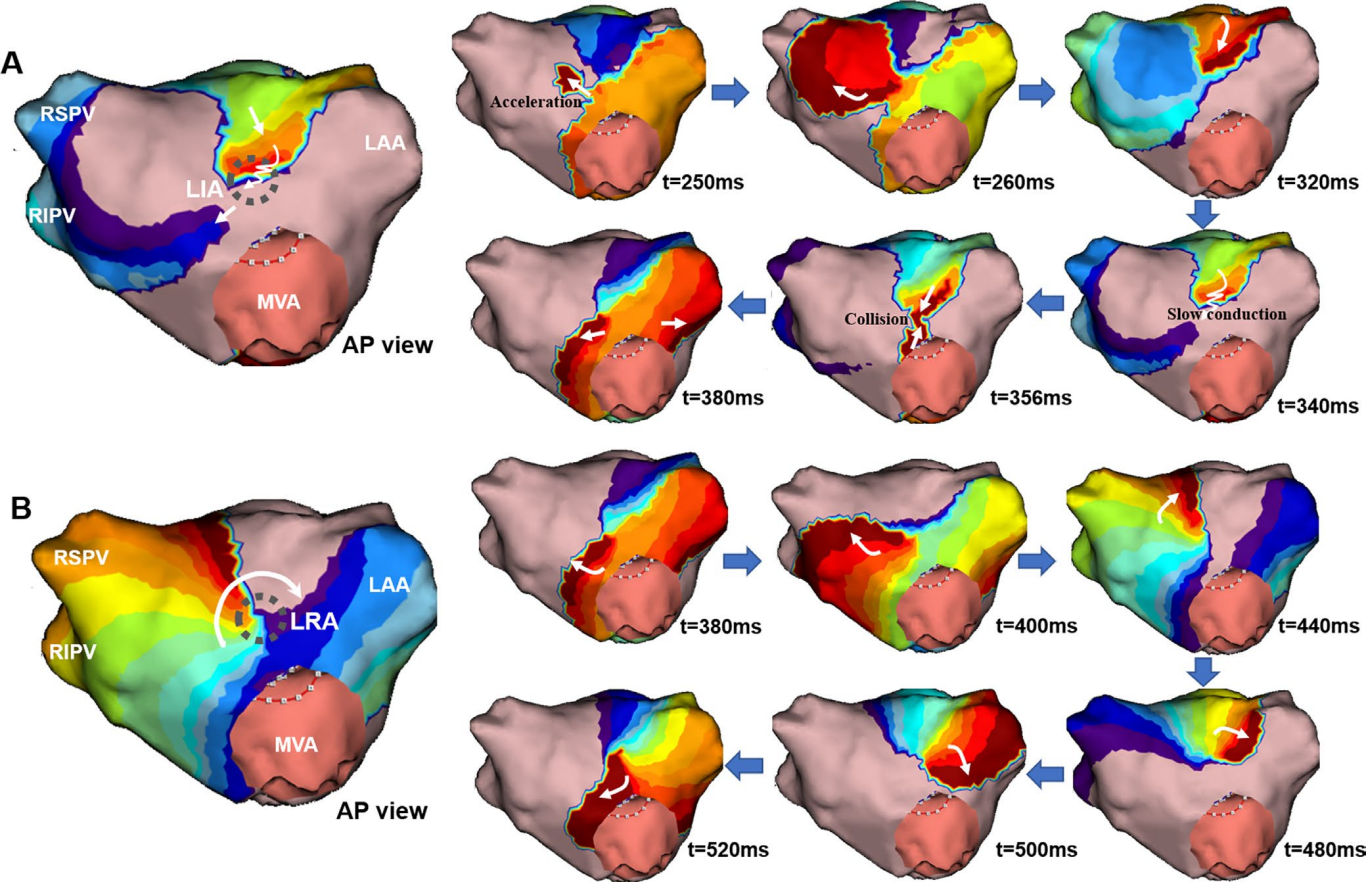
Preferential conduction area showing two consecutive activation patterns in the same patient. Panel A shows a localized irregular activation (LIA) detected on the mid‐anterior wall of the left atrium. Panel B shows a clockwise localized rotational activation (LRA) which subsequently occurred in the same region within the same R‐R interval. The grey circle represents the preferential conduction area. The time (milliseconds) at the bottom of each snapshot represents the moment when the snapshot is taken. White arrows delineate the propagation directions of the activation patterns

Although appearing initially random and variable, the activation patterns were confined to specific preferential conduction areas, on average 4 ± 2 LA regions per patient. These were most commonly identified in the mid‐anterior (48%) and lower‐posterior (40%) walls where the dominant activation patterns were LRA and LIA. Figure 7 shows an example of a preferential conduction area on the mid‐anterior wall. During this short period, LIA was detected on the mid‐anterior wall with a combination of slow conduction and collision, followed by a LRA in the same region. The identified activation patterns repeated at preferential conduction areas in each case.

## 4 DISCUSSION

### 4.1 Main findings

This study is the first to characterize localized activation patterns of persistent AF identified from noncontact charge‐density mapping. The main findings of this study are as follows:

1. Persistent AF is characterized by highly dynamic and heterogeneous patterns of atrial activation consisting predominantly of LIA (63%). LRA and FCA accounted for 20% and 17%, respectively.
2. LIA occurred in all LA regions, but was more common on the anterior and posterior walls.
3. LIA consisted of a combination of slow conduction, pivoting, collision, and accelerated conduction.
4. On average, 4 ± 2 preferential conduction areas were observed per patient with highest frequency of repetitive LRA and LIA commonly observed in the mid‐anterior (48%) and lower‐posterior (40%) walls.
5. The coalescence of LIA into a single broad wavefront can result in LRA and sustained LRA may break and degenerate into LIA.

### 4.2 Mechanisms of AF

While the PVs are known to be triggers for the initiation of paroxysmal AF,^1^ the mechanisms that are responsible for the perpetuation of persistent AF in humans are controversial and likely to vary between individuals. Existing hypotheses propose that single or multiple localized stable (focal/reentrant) sources or multiple unstable wavelets may drive or maintain AF.^13^, ^14^ High‐resolution contact mapping studies in animals and patients characterizing wavefront propagation during AF also support the notion that multiple wavelets giving rise to fibrillatory conduction are associated with decreased atrial refractory period and heterogeneous tissue structure.^15^ Recent experimental and clinical studies have led us to revisit the determinant role of drivers (discrete foci or reentry) during AF.^16^ Jalife and colleagues used phase‐based optical mapping to show that AF is organized by one or a small number of high‐frequency rotating sources localized to the LA.^17^ The increased dimension of fibrosis (substrate) was associated with rotor facilitation as well as the complexity of propagation.^18^

Drivers and maintainers are difficult to detect in persistent AF using conventional sequential mapping because of intermittent firing and spatial meandering. Theoretically, panoramic global mapping can help to identify the characteristics of AF activation and provide a better understanding of the hierarchy of the above‐mentioned mechanisms responsible for AF perpetuation. This underpins the merit of using such mapping systems to characterize dynamic AF activation patterns.

### 4.3 Human global AF mapping

Using contact endocardial basket mapping, Narayan et al reported AF persistence owing to temporally stable rotors or “drivers”.^5^ However, other studies using panoramic epicardial or endocardial mapping have observed predominantly unstable rotors.^6^, ^19^, ^20^ Cuculich et al reported that rotors were seen rarely (15%) using noninvasive body surface mapping with the most common patterns of AF being multiple wavelets (92%) and foci (62%).^6^ Pathik et al observed that transient rotational activity was detected in 64% of patients using 3D phase mapping, but 44% of patients formed them at the same location.^20^ These intermittent and spatially unstable drivers were reported to be clustered in structurally heterogeneous tissue that provided a substrate for perpetuation of persistent AF.^16^

The AF patterns detected by charge‐density mapping were notably complex and varied from cycle to cycle. Three main activation patterns were observed during persistent AF. The most common patterns were LIA (63%) and LRA (20%) with varying degrees of interaction between these two. The FCA and LRA patterns observed in the present study are generally consistent with the concept of driver sites described in previous studies.^6^, ^17^ While the basic electrophysiological mechanism of LIA is not clear, we postulate that it may be because of dynamic spatial dispersion of atrial refractoriness. It represents fibrillatory conduction at one end, and pivoting, wavefront propagation through an isthmus (acceleration) as well as source‐sink mismatch in other aspects. Further studies on delineating anatomical areas of LIA would offer the opportunity to determine whether it is of mechanistic importance or a bystander phenomena of AF as shown in the latest results of panoramic AF recordings.^21^

### 4.4 Interaction of mapped mechanisms

It is noteworthy that coalescence of LIA into a single broad wavefront can result in LRA and conversely, sustained LRA may break and degenerate into LIA. This implies that organized rotational activation can give rise to complex irregular patterns of excitation, whereas sustained LIA may also result from uninterrupted periodic activity of LRA. Kowalewski et al reported organized AF patterns waxed and waned because of competition from other organized AF sites or fibrillatory waves, before reappearing in similar spatial locations.^22^ Further studies are warranted to define the mechanisms of interplay between activation patterns and whether knowledge of such mechanisms may improve mapping and ablation of persistent AF.

We propose that persistent AF is driven and maintained by the coexistence of these characteristic activation patterns with each pattern influencing the other depending on the anatomic and functional properties of the underlying substrate. The present study is the first to characterize complex components of LIA formed by a combination of multiple activations of slow conduction, pivoting, collision, and acceleration. Konings et al observed that slow conduction, pivoting, and collision were associated with fractionated unipolar electrograms, indicating that these activation patterns were associated with dyssynchronous activation in the atrial wall.^23^ The relative contribution of each component to the initiation and maintenance of LIA remains to be determined.

The frequency of FCA observed in the present study was relatively low (17%), originating predominantly from the PVs/ostia. This is consistent with the frequency and location of FCA sites from an earlier AF mapping study,^6^ thus highlighting the relevance of PV ostia in some persistent AF. Some instances of apparently focal activation may stem from intramural conduction in the human atria projecting obliquely on the endocardial and epicardial surfaces.^24^ Endocardial or epicardial breakthrough may appear as focal activity that actually arose from fibrillatory waves conducting between dissociated epicardial layers of the atrial wall.^25^

### 4.5 Preferential conduction areas

In the present study, the activation patterns were varying and transient but repetitive at certain preferential locations. This was consistent with findings from Kowalewski et al who demonstrated disordered AF activation yet with regions of spatial organization. There may be more than one such organized site competing and resulting in the destabilization of repetitive activation patterns.^22^ We found the most common sites of LIA and LRA were anchored at the mid‐anterior and lower‐posterior walls. This supports the notion that sites with complex fiber orientation and increased interstitial fibrosis may provide important substrates for AF maintenance because of the anisotropic atrial tissue conduction.^8^, ^24^, ^26^ Repetitive patterns of activation at preferential conduction areas suggest LIA and LRA may share a common underlying tissue substrate or functional coupling governed by a substantial range in dispersion of refractoriness within and adjacent to these areas, whereby one promotes the formation of the other in the vicinity.^8^, ^16^ Similarly, anatomical and functional characteristics of the regions of substrate between identified areas may macroscopically modulate the spatiotemporal sequence of activation patterns. Thus, our hypothesis is that these preferential conduction areas with repetitive activation patterns may be potential ablation targets for treating AF.

### 4.6 Clinical implications

Despite advances in imaging guidance, catheter technologies, and variations in ablation strategy, the long‐term outcome from catheter ablation of persistent AF remains low when compared with paroxysmal AF. A better understanding of patient‐specific mechanisms of AF may improve risk stratification and patient selection for ablation. An understanding of the mechanistic coexistence of focal, rotational, and irregular activation in persistent AF and their spatiotemporal coupling may explain the variability in clinical outcome and promote individualized ablation strategies to treat persistent AF.

Observations from the present study suggest strategies should target both triggers and maintainers given the combination of different activation mechanisms in persistent AF. An individualized ablation strategy may improve outcome using an iterative approach of PV isolation first, followed by individualized target of focal sites of non‐PV triggers and then LRA/LIA prioritized by the dominance of the activation patterns and specific areas of preferential conduction with an aim to stabilize or abolish reentry activities.^12^ Further studies are needed to characterize the respective contribution of each activation pattern in human AF and the progression of the structural substrate during AF continuation. Detection of zones with consistent and repetitive activation patterns will be a key factor in optimizing a patient‐specific ablation strategy.

### 4.7 Limitations

Epicardial activation was not evaluated simultaneously, thus we cannot distinguish focal activation from an epi‐ or endo‐breakthrough site. We were not able to perform simultaneous biatrial mapping and therefore could not draw any conclusion on the relationship between the activation patterns between the two atria. Longer durations of AF activation maps may provide further insights into the temporal distribution of these characteristic activation patterns. The role of 3D structure and complex geometry of the atrial myocardium on the different types of activation was not evaluated. Studies assessing the clinical outcome using individualized ablation strategies guided by noncontact charge‐density mapping are needed.

## 5 CONCLUSION

Human persistent AF is characterized by a notably heterogeneous and coexistent variety of activation mechanisms identified in PV‐ostial and non‐PV regions throughout the LA. LIA and LRA are the most common activation patterns with varying degrees of interaction between them. The clinical implications of individualized ablation strategies guided by charge‐density mapping need to be determined.

## AUTHOR CONTRIBUTIONS

All authors contributed to research design, analysis of the data, drafting or revising, and approval of the manuscript.

## Supporting information

Supplementary Material
