# Supplementary material for: Diverse activation patterns during persistent atrial fibrillation by noncontact charge‐density mapping of human atrium
Source: J Arrhythm. 2020 May 20;36(4):692–702. doi: 10.1002/joa3.12361 (PMC7411208; doi:10.1002/joa3.12361)
Supplement: Supplementary file 1 — Supplementary Material [file JOA3-36-692-s001.docx]

**SUPPLEMENTAL MATERIAL**

**Legends for the Movie files**

**Movie A**. An example of a focal centrifugal activation (FCA) involving the left inferior pulmonary vein (LIPV) in a patient with persistent AF. White arrows show the centrifugal path of the propagation around the LIPV. LAA = left atrial appendage; LIPV = left inferior pulmonary vein; LSPV = left superior pulmonary vein; RIPV = right inferior pulmonary vein; RSPV = right superior pulmonary vein.

**Movie B**. An example of localised rotational activation (LRA) on the lower-posterior wall of the LA in a patient with persistent AF. The white arrow shows the rotational direction of the LRA. LIPV = left inferior pulmonary vein; LSPV = left superior pulmonary vein; RIPV = right inferior pulmonary vein; RSPV = right superior pulmonary vein.

**Movie C**. An example of localised irregular activation (LIA) on the posterior wall of LA in a patient with persistent AF. The white arrows show the activation direction of the LIA. A pivot can be seen first, then wave front collision, followed by slow conduction and then acceleration through a gap at the posterior wall. LIPV = left inferior pulmonary vein; LSPV = left superior pulmonary vein; MVA = mitral valvular annulus; RIPV = right inferior pulmonary vein; RSPV = right superior pulmonary vein.

**Methodology (supplement material)**.

**Charge-density principle and interpretation of the propagation map**

The charge-density principle and interpretation of the propagation map underlies the clinical relevance and effectiveness of this novel mapping technology. At the cellular level, microscopic dipoles (closely spaced positive and negative ionic charges) emerge and disappear from the action of ion channels. As propagation ensues, the dipoles act as a macroscopic double layer of charge-density (Coulombs/cm) that embodies the wave front and generates the cardiac potential-field (Volts) across and throughout the torso volume. Accordingly, charge-density is a more localised entity that represents the actual biophysics of cardiac activity at the tissue level and provides a more focused view into the details of cardiac activation. The fundamental difference between voltage and charge-density lie in both the averaging effect of ‘spatial summation’ and in the volume of space occupied by each.

The AcQMap System employs an inverse solution based on Poisson’s equation to derive the dipolar charge sources that exist within the myocardium. Poisson’s equation relates the distribution of sources of charge (i.e. charge density) to the surrounding distribution of voltage that the sources generate. Accordingly, the voltage at any such point is expressed as:

$V\left( \vec{x} \right)=\int_{S} d\left( \vec{y} \right)\frac{cos\varphi_{xy}}{\left| \vec{x}-\vec{y} \right|^{2}}d\sigma_{y}$

Where $V(\vec{x})$ is the voltage at any point within the chamber; and

$\int_{S} d\left( \vec{y} \right)\frac{cos\varphi_{xy}}{\left| \vec{x}-\vec{y} \right|^{2}}d\sigma_{y}$ represents the summation of dipolar charge sources within the myocardium$.$

The inverse algorithm relates the 48 points of measured voltage on the AcQMap catheter electrodes (known values on left-side of equation) to a set of 3648 ‘equivalent’ dipolar sources on the chamber surface (unknown values on right-side of equation). The matrix of values is regularized and inverted to derive the local charge-density across the chamber surface. The distribution of charge-density is calculated at every sample-time to attain a global, temporal ‘playback’ of propagation of charge-density across chamber anatomy.

Solving the inverse solution requires three key inputs: 1) multiple simultaneous measurements of the cardiac potential-field in the chamber, which can be collected by the 48 electrodes on the AcQMap catheter; 2) an accurate anatomic surface (ultrasound anatomy construction) used to define the location of the charge sources; and 3) a common coordinate system in which the AcQMap catheter is localised with 3D coordinates that correspond with the reconstructed anatomical mesh. Provision of the inputs listed above enables spatially-localised and temporally-animated derivation of the charge-density on the endocardial surface. The corresponding waves of activation are displayed across the reconstructed 3D surface through time.

The activation wave front can be displayed in its rawest form as either the magnitude of charge-density or voltage, with voltage forward-calculated from the derived charge-density. Additional postprocessing of the data can be applied to extract specific information from the map. Maps depicting activation are constructed from activation-times derived at each vertex of the chamber anatomy, using a spatio-temporal method. Specifically, all charge-density waveforms (charge-density values at each vertex through time) are normalised by the maximum peak-negative value from all the waveforms. Then, the temporal derivative of each waveform and the spatial Laplacian at each vertex (for each time-instant) are computed from the normalised charge-density values. The instant-of-activation for a charge-density waveform is determined using a set of logical rules with relative (normalised) thresholds:

1. Mark candidate activation-times through the duration of each waveform at every instant when the Laplacian is: (a) at least 1% of the magnitude of the max Laplacian amplitude; and (b) has a negative temporal derivative.
2. Identify the nearest zero crossing in the waveform on the same temporal derivative downslope determined in 1).
3. Exclude candidate activation-times with peak-to-peak amplitudes of the waveform that are less than 2% of normalised signal (This parameter is user adjustable).
4. Choose the activation-time with the most negative temporal derivative if multiple candidate activation-times are detected within a user-adjustable refractory period. All other candidate activation-times within the refractory period are discarded. The refractory period is usually set to ~75% of the cycle-length for a ‘regular’ rhythm or the average fibrillatory cycle-length for AF.

Using the above criteria, a final set of local activation-times are selected and a ‘propagation history’ map is generated that delineates the location of the leading edge of the wave front at steps in time across the duration of a user-adjustable history-window. The duration of history is set to a physiologically relevant level that is less than the rhythm cycle-length. Like the refractory period, this is usually set to ~75% of the rhythm cycle-length (or average fibrillatory cycle length).

A propagation-history map uses bands of colour to show the location and velocity of the leading edge of the wave front over a set duration of time. The red colour-band represents the leading edge at the present instant of time, while the other colours represent the location of the leading edge at earlier steps in time. The width of the colour bands conveys the conduction velocity of the wave front, with wider bands indicative of fast conduction and narrow bands of slow conduction. Ultimately, animated playback of the propagation history map enables a clear understanding of regular, irregular, and complex patterns of activation.

**Line of block identified by charge-density mapping**


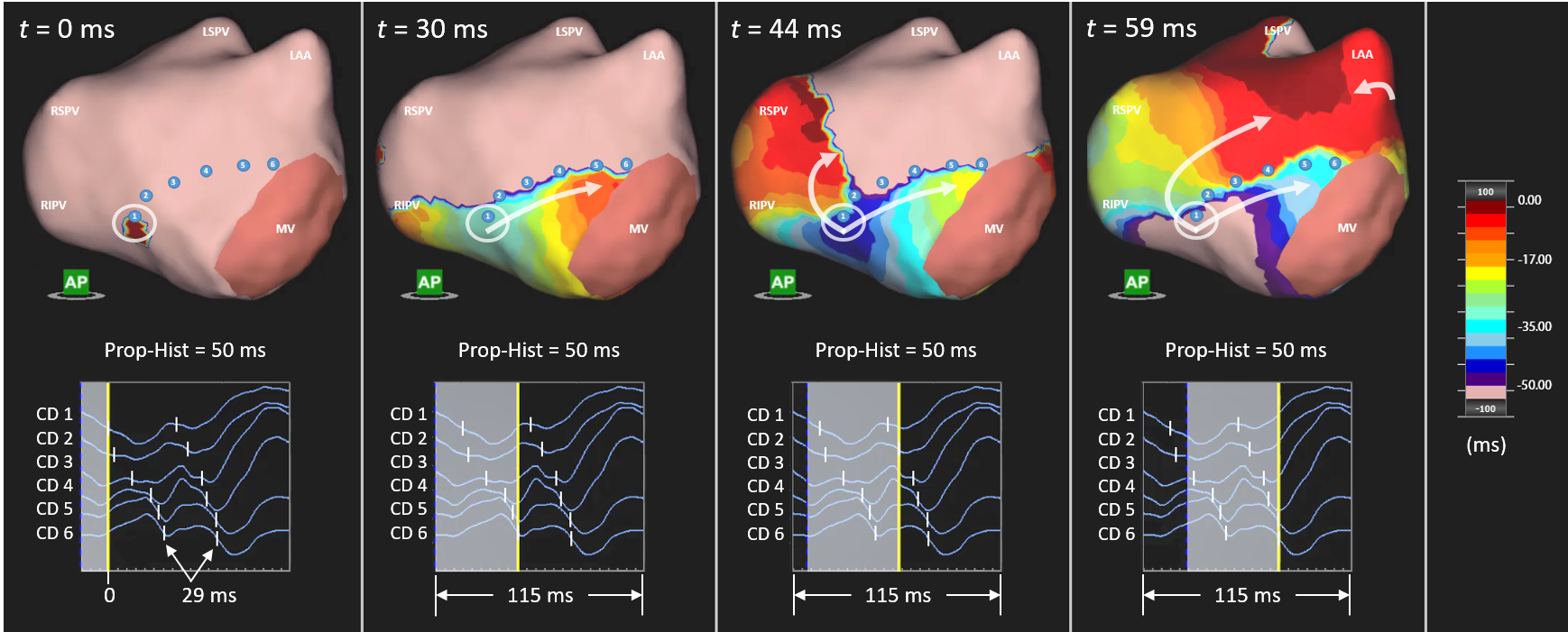


*The figure panels above show an organised wavefront breaks through on the septum and propagates around both sides of the line-of-block on the anterior wall in the left atrium. Four still propagation images show the progression of isochronal activation on the anatomical map. The split deflections in signals reconstructed along a line-of-block (CD1 to CD6) are associated with the delayed pattern of isochronal activation displayed on the anatomical map.*
